# Rapid emergence of non-autonomous elements may stop *P*-element invasions in the absence of a piRNA-based host defence

**DOI:** 10.1371/journal.pgen.1011649

**Published:** 2025-08-20

**Authors:** Matthew Beaumont, Divya Selvaraju, Riccardo Pianezza, Robert Kofler

**Affiliations:** 1 Institut für Populationsgenetik, Vetmeduni Vienna, Wien, Austria; 2 Vienna Graduate School of Population Genetics, Vetmeduni Vienna, Vienna, Austria; Cornell University, UNITED STATES OF AMERICA

## Abstract

Transposable element (TE) invasions pose risks to both the TE and the host. All copies of a TE may be lost via genetic drift, or host populations may suffer fitness declines, potentially leading to extinction. By monitoring invasions of the *P*-element in experimental *D. melanogaster* populations for over 100 generations, we uncovered a novel risk for invading TEs. In two replicate populations, the *P*-element rapidly multiplied until a piRNA-based host defence emerged, leading to the plateauing of TE copy numbers. However, in one population (R2), *P*-element copy numbers stabilised at a significantly lower level, despite the absence of a piRNA-based host defence. We find that this stabilisation was likely driven by the propagation of non-autonomous insertions, characterised by internal-deletions, which out-competed the autonomous full-length insertions. Such a rapid proliferation of non-autonomous insertions could account for the high prevalence of *P*-element insertions with internal-deletions observed in natural *D. melanogaster* populations. Our work reveals that TEs may stochastically sabotage their own spread in populations due to the emergence of non-autonomous elements, rendering the establishment of a host defence unnecessary. The proliferation of non-autonomous elements may also lead into an evolutionary dead end, where affected populations are resistant to re-invasion (*e.g*. following recurrent horizontal transfer), yet are unable to infect other species due to a lack of autonomous insertions.

## Author summary

Transposable elements (TEs) are short, self-replicating DNA sequences found in nearly all genomes. While they can be harmful to their hosts, many organisms have evolved defence systems, most notably utilising piwi-interacting RNAs (piRNAs), to suppress their activity. In this study, we introduced the DNA transposon *P*-element into three replicate populations of the fruit fly *D. melanogaster* to study how TEs invade and how hosts respond. Surprisingly, in one population, the *P*-element invasion stalled despite the absence of an active piRNA response. We show this was likely due to internal deletions arising early in the invasion, caused by the element’s own faulty replication. Our results highlight a previously underappreciated outcome of TE invasions: failure due to internal instability, without the need for host-mediated repression.

## Introduction

Eukaryotic organisms have long faced the threat of transposable element (TE) invasions. These stretches of DNA integrate into host genomes and selfishly replicate, irrespective of fitness effects [16,57]. TEs have proven extraordinarily effective in self-transmission, having been able to invade almost all observed eukaryotic genomes [6,87]. They show varying success rates in colonising different species, where the total TE content in host genomes ranges from just 3% in yeast, to 78% in Antarctic krill [6,75]. Although some TE insertions have been posited to be beneficial to a host [2,21], it is generally assumed that most insertions are either neutral or deleterious. Left unchecked, the ever-propagating TE poses a threat to genome stability and potentially even population survival as a whole [36,37,55]. In response, hosts have developed intricate defence mechanisms by which they can limit replication, typically utilising small RNAs [70]. In *Drosophila*, host defence is based around piRNAs, small RNAs ranging in size from 23 to 29 nt that silence TEs at both the transcriptional and post-transcriptional levels [10,24,43,77]. These piRNAs are derived from distinct genomic loci, termed piRNA clusters, comprising around 3.5% of the total genome in *D. melanogaster* [90]. A fundamental component of piRNA biogenesis is the ping-pong cycle, which amplifies the abundance of piRNAs through a positive feedback loop involving two cytoplasmic proteins, Aub and AGO3 [10,24]. Cleavage of TE transcripts by Aub yields novel piRNAs, which may then be loaded into AGO3 to guide the cleavage of further transcripts into piRNAs, that are then again loaded into Aub. Slicing of TE transcripts also brings about ’phasing’, in which the resulting piRNA precursors are processed by the endonuclease, Zuc [15,25]. Whilst the ping-pong cycle amplifies piRNA abundance, phasing is thought enhance piRNA diversity [15,25,51]. It is unclear what initially triggers the emergence of a piRNA-based host defence [20,46,71]. The current prevailing model, the trap-model, holds that an invading TE is stopped when it transposes into a piRNA cluster, which then triggers the production of piRNAs that suppress the TE [5,48,58,91].

Another mechanism that may affect the proliferation of a TE, are non-autonomous insertions. Non-autonomous elements were first described by Barbara McClintock as the now famous pair of loci, Ds and Ac, controlling chromosomal breakage in maize [50]. Later, it was discovered that Ac is a full-length autonomous TE and Ds a non-autonomous element, whose activity depends on Ac [19]. These non-autonomous insertions are unable to produce the proteins necessary for mobilisation, but can utilise the proteins generated by autonomous insertions [27]. Non-autonomous insertions have been observed for many TE families. It is possible that some non-autonomous elements benefit from a mobilisation advantage over autonomous insertions. For *Mariner* in *D. melanogaster*, the non-autonomous element (*Peach*) seems to proliferate more efficiently than the autonomous (*Mos1*). A notable example of these dynamics, and focus of this study, are the non-autonomous *P*-elements in *Drosophila* species. The *P*-element is a 2907 bp DNA transposon with four ORFs [7] that was famously discovered as the causative agent of hybrid dysgenesis, where the offspring of reciprocal crosses among the same two strains may show varying ovarian phenotypes (i.e. atrophied vs regular) [7,34]. The *P*-element is active in the germline but not in the soma [41], theorised to be a strategy to minimise damage to the host [12]. This tissue specificity is regulated by alternative splicing of the third intron (IVS3), which is spliced out in the germline but retained in the soma [41]. Interestingly, the piRNA-based host defence acts by repressing IVS3 splicing in the germline [83]. Internal deletions (IDs) of the *P*-element have been frequently observed in prior work [8,18,38,39,74]. Many of these *P*-element insertions with IDs are non-autonomous and may be mobilised by the transposase produced from full-length insertions. Some may be preferentially mobilised relative to their full-length copies [28,29,39,82]. Interestingly, certain non-autonomous *P*-element insertions, like the *KP*-element or *D50*, generate proteins that may even act as repressors of *P*-element activity [8,65]. Non-autonomous *P*-element insertions can yield defective transposase proteins that are able to occupy transposase binding sites, thereby blocking access of functional transposases preventing mobilisation [45]. Importantly, the regulation of TEs by non-autonomous elements is not a feature of the host defence but rather a limitation of the mechanism by which TEs replicate. Irrespective of which method controls TE activity, inactive TE families will gradually accumulate mutations that will render all TE copies non-functional. Such inactive TEs will therefore eventually face extinction [9]. To escape this gradual erosion by mutations, TEs occasionally undergo horizontal transfer (HT) into a novel unprotected species, where they are able to replicate until they are once again silenced by the host. Such TE invasions triggered by HT may be far more common than previously thought [59,60,71]. However, the invasion of novel species may pose some risk for the newly arrived TE as well as to the host. First, even after successful HT into a novel species, a TE may fail to become established in a host population [42]. All copies of the TE may be lost due to drift or negative selection. Second, TE invasions could dramatically reduce the fitness of the host, such that the survival of the host population is threatened [36,37]. For example, we previously found that the establishment of the piRNA-based host defence may fail stochastically in populations invaded by the *P*-element, with dramatic effects on host fitness [74]. A startling decline in host fitness that eventually led to the extinction of the experimental population has also been seen in other works [84]. Extinction of host populations will, of course, also all remove all active TEs copies. Assuming that each TE only has but a few opportunities to spread by HT to a novel species before gradual erosion by mutations deactivates all copies, it is crucial for the TE to efficiently utilise these limited opportunities, or otherwise face extinction.

To shed light on the evolutionary dynamics of TEs we monitored *P*-element invasions in experimental *D. melanogaster* populations. Earlier studies monitoring the spread of the *P*-element in experimental populations found that the *P*-element may spread into naïve genomes [22,33,52]. Contemporary studies have focused more on the interaction between the *P*-element and the piRNA-based host defence. For example, [32] suggested that insertions in piRNA clusters trigger suppression of the *P*-element. Multiple recent works show that piRNAs complementary to the *P*-element are typically emerging within just a few generations after introducing the *P*-element into a naïve population [38,39,74,84]. Interestingly, in a single replicate population of one study, no piRNA-based host defences emerged despite although the *P*-element attained high copy numbers and multiple insertions in piRNA clusters were found [74]. In a single replicate of another study, the *P*-element was at low copy numbers after 52 generations, despite the presence of full-length insertions and the absence of piRNAs [84]. However, it is unclear if the *P*-element was ever active in this replicate.

By monitoring *P*-element invasions in three experimental *D. melanogaster* populations for 100 generations, we discovered a novel threat to the long-term persistence of TEs. The *P*-element spread rapidly in two (R1, R3) out of the three replicates, where the emergence of a piRNA-based host defence led to stable copy numbers between generations 30-40. However, in one replicate (R2) copy numbers stabilised at around the same time at a significantly lower level, despite the absence of a piRNA-based host defence. *P*-element insertions with IDs, likely non-autonomous elements, proliferated in this replicate to such an extent that few autonomous copies remained, likely resulting in the stabilisation of *P*-element copy numbers. However, our work reveals a novel risk of TE invasions, i.e. that non-autonomous IDs may rapidly emerge during an invasion and spread such that few autonomous copies remain. Such a proliferation of non-autonomous elements may lead into an evolutionary dead end, endangering the long-term persistence of TEs.

## Results

### *P*-element invasions in experimental *D. melanogaster* populations

**Fig 1:**
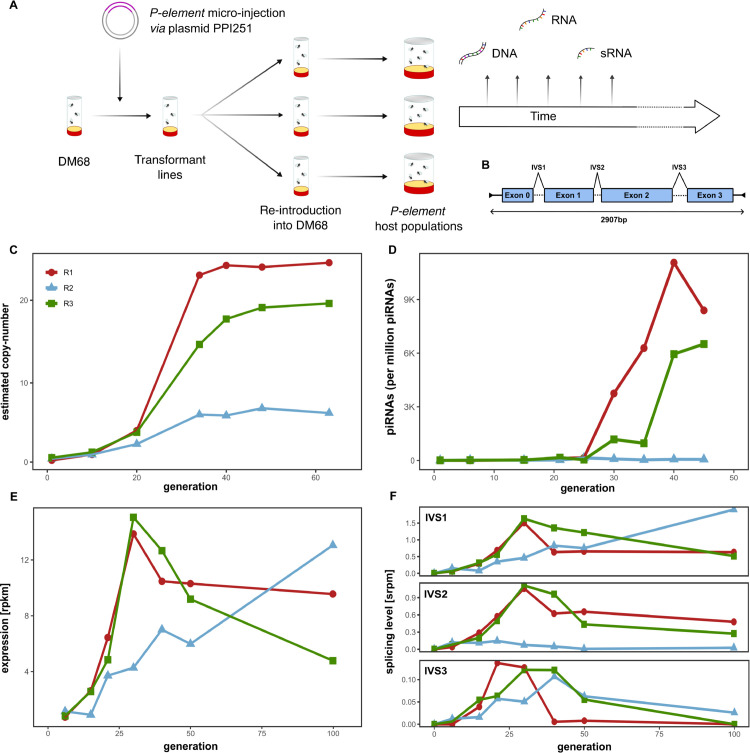
*P*-element invasions in three experimental D. melanogaster populations (R1, R2, R3). **A** Overview of our experimental design. **B** Basic schematic of the *P*-element, including exons, intervening sequences (IVS) and TIRs (black arrows). **C** *P*-element copy numbers in each replicate across time. **D** piRNAs mapping to the *P*-element across time. **E** Expression of the *P*-element across time. **F** Abundance of spliced reads for each *P*-element intron (IVS1-3) in spliced reads per million (srpm).

To study the dynamics of TE invasions, we introduced the *P*-element, via micro-injection, into a *D. melanogaster* strain (DM68) without any *P*-element insertions (S1 Fig). We then established three replicate populations (R1, R2, R3), by mixing transformed flies with naïve DM68 flies. Populations were maintained at a size of *N* = 250 and a temperature of 25° C, with non-overlapping generations. We monitored the following *P*-element invasion in each population for more than 100 generations. At regular time intervals, we used short-read sequencing on pooled genomic DNA from each replicate, as well as the transcriptome (Fig 1A). We used the tool DeviaTE [85] to estimate the *P*-element copy number in each sample, which normalises the coverage of TEs to the coverage of single-copy genes (Fig 1C). For example, if the *P*-element has a coverage of 50x and the single-copy genes an average coverage of 5x, then we infer 10 *P*-element copies per haploid genome.

We observed that *P*-element copy numbers increased in all replicates until generations 35-40, where copy numbers reached a stable plateau (Fig 1C). In R1 and R3, this plateau was around 20-26 copies per haploid genome, whereas in R2, copy numbers plateaued at a much lower level of ∼7 copies. We then asked what could be responsible for the lower level observed in R2. We first tested whether the piRNA-based host defence was established faster in R2 than in the other replicates. A rapid emergence of a host defence could limit the accumulation of *P*-element copies. To assess this, we investigated the abundance of small RNAs mapped to the *P*-element in the experimental populations. Contrary to our expectations, we found that a large number *P*-element piRNAs emerged around generation 25-40 in R1 and R3 but not in R2 (Fig 1D). In addition, the number of siRNAs was very low in R2 (S2 Fig). This raises the important question as to how the invasion was so effectively cut short without a piRNA-based host defence. Next, we wondered whether the *P*-element was silenced via another mechanism, independent of piRNAs. To test this, we sequenced the bulk mRNA of each pooled sample. *P*-element expression increased in all replicates until generation 30 (Fig 1E). In R1 and R3, expression then slowly declined. Contrastingly, *P*-element expression in R2 increased continuously over time, reaching it’s highest value at generation 100 (i.e. the latest available time point; Fig 1E). Therefore, our data suggests that the *P*-element transcription was not repressed in R2.

Splicing of the *P*-element introns is essential for it’s biology [1,76]. IVS3 particularly so, as the tissue specificity of the *P*-element is regulated by alternative splicing of this intron (IVS3 is spliced out in the germline but retained in the soma [41]). Additionally, the piRNA-based host defence silences the *P*-element by repressing splicing of IVS3 [83]. We estimated the splicing level of all three introns of the *P*-element during our experiment (Fig 1F). In R1 and R3, the splicing of all three introns decreased around generation 20-30, where the level of splicing of IVS3 was most dramatically reduced. By contrast, we still found high levels of splicing of IVS3 at generation 100 in R2 (Fig 1F). Here, splicing of IVS2 also remained at a constantly low level throughout, in contrast to the levels of IVS1 and IVS3 splicing, that either increased or fluctuated during the experiment (Fig 1F).

Together, these results show that in two replicates (R1 and R3), *P*-element copy numbers increase to approximately 20 copies per haploid genome and then stabilise as piRNAs emerge. This stabilisation in copy numbers coincides with reduced *P*-element expression and splicing of IVS3. In one replicate (R2), copy numbers plateau at a significantly lower level, despite lacking a piRNA-based host defence. *P*-element expression in R2 also continues to increase and IVS3 splicing is still observed after 100 generations.

### Inactive ping-pong cycle in R2

**Fig 2:**
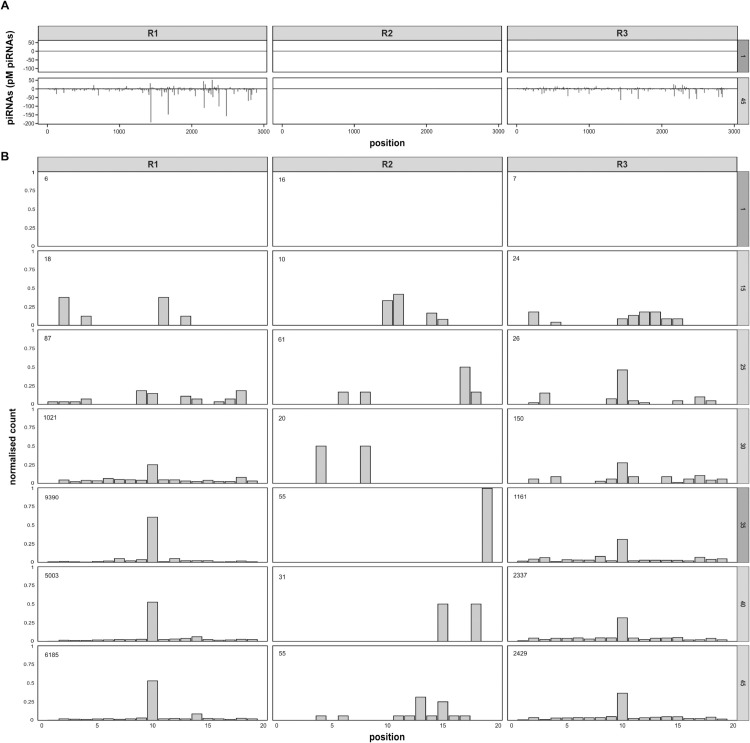
The low abundance of *P*-element piRNAs in R2 may be due to an inactive ping-pong cycle. **A** Distribution of piRNAs on the *P*-element at generation 1 and 45 (right panel). Sense piRNAs are shown on the positive y-axis and antisense on the negative. Samples from the whole-body of female flies are labelled in a light-grey and those taken from ovaries are in dark-grey. **B** Ping-pong signatures for the *P*-element in different replicates during the experiment (generations denoted in the right panel). Values in the top-left corner show the total number of *P*-element piRNAs in the sample.

We next investigated in more detail as to why so few *P*-element piRNAs were generated in R2. In R1 and R3, small RNAs mapping to the *P*-element are primarily between 23-29 nt long, and largely have a ’U’-bias at the first base, as expected of piRNAs (S3 Fig [10]). However, in R2, small RNAs mapping to the *P*-element predominantly have a size of 21 nt, alongside a less pronounced U-bias than both R1 and R3, more indicative of siRNAs than piRNAs (S3 Fig; [15]). At later generations, piRNAs are broadly distributed over the *P*-element in R1 and R3 and almost entirely absent in R2 (Figs 1D, 2A and S4 Fig). piRNA abundance is thought to be amplified by the ping-pong cycle, a positive feedback loop, wherein the cleavage of sense and antisense transcripts of TEs results in novel piRNAs [10,44]. Therefore, we questioned whether the ping-pong cycle was inactive for the *P*-element in R2. An active ping-pong cycle leads to a distinct signature; piRNAs produced from opposing strands will frequently overlap by 10 nt at the 5’ ends, termed the ping-pong signature [10,24]. We found a ping-pong signature emerging for the *P*-element between generations 25-30 in R1 and R3, but we could not observe a ping-pong signature at any generation in R2 (Fig 2B and S5 Fig). However, the ping-pong cycle is functional in R2, as we found a clear signature for another TE (Blood) (S6 Fig). Downstream of the ping-pong cycle, an additional process termed ’phasing’ may be active, in which cleaved piRNA precursors are further processed into piRNAs by the endonuclease Zucchini [15,25,51]. Phasing also leads to a characteristic pattern in the distribution of the distance between the 5’-end and 3’-start of neighbouring piRNAs, where a distance of 1 nt is overrepresented [25]. We observe this signature for *P*-element mapping piRNAs at later generations in R1 and R3, but have too few piRNAs to compute it in R2 (S7 Fig). Interestingly, we found at least one piRNA cluster insertion within each replicate, including R2 (S14 Fig, [79]). To summarise, the absence of the ping-pong cycle for the *P*-element likely accounts for the low abundance of piRNAs in R2.

### Rise of non-autonomous *P*-element insertions in R2

**Fig 3:**
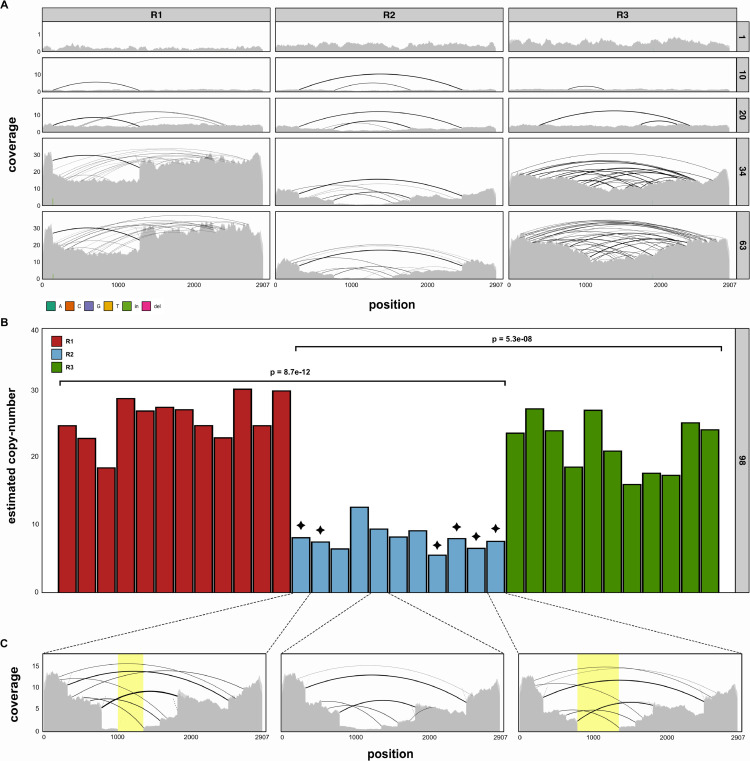
Overview of structural variation in the *P*-element during the experiment. **A** Abundance of *P*-element insertions and IDs across all three replicates (top) at different generations (right panel). Plots show the coverage normalised to single-copy genes, and the positions of IDs (inferred from split-reads) as arcs. **B** *P*-element copy numbers for 11-12 individual flies, sampled at generation 98. Significant differences between replicates are shown at the top (t-tests). Black stars denote individuals without a single full-length insertion. **C** *P*-element coverage of selected individuals from B. Yellow highlighted regions show areas with zero coverage. Individuals with zero-coverage regions cannot contain a single full-length *P*-element insertion.

**Fig 4:**
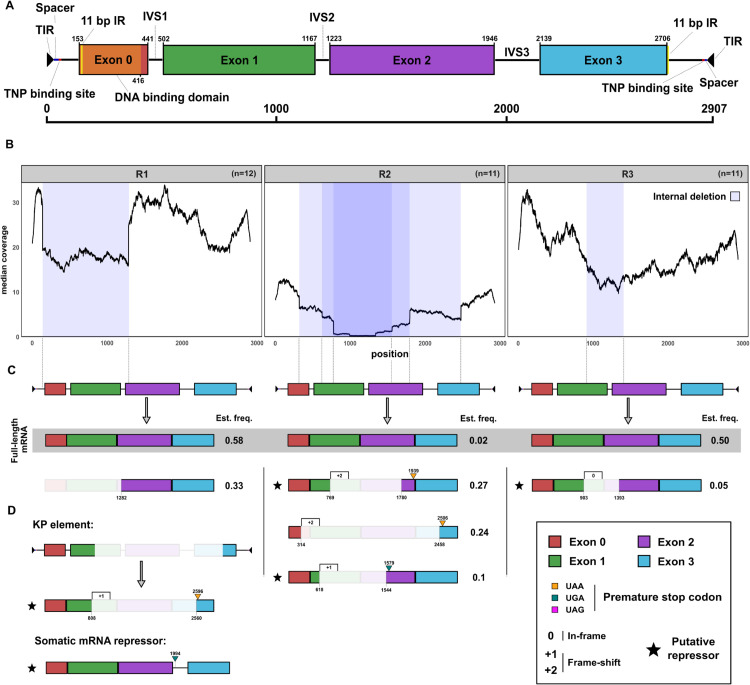
Proteins generated by *P*-element insertions with IDs in each replicate. **A** Schematic of the structure of the full-length *P*-element, highlighting the DNA binding domain and sites necessary for the mobilisation of the *P*-element (transposase binding sites, TIRs) [45,47]. **B** Average coverage of the P-element across individuals in each replicate population, shaded blue regions denote prominent IDs (frequency ≥0.05) [85]. **C** Transposases encoded by different *P*-element insertions in our experimental populations. Their estimated frequency is shown on the right side. Premature stop-codons (yellow triangle) and frame shifts (rectangle with number) are highlighted. Putative repressors are highlighted by a star. **D** Schematic representation of the somatic mRNA repressor of the P-element and the *KP*-element (and its mRNA) [8,67].

*P*-element activity can be regulated by non-autonomous insertions with IDs, such as the *KP*-element [8]. Proteins produced from such defective insertions may, for example, occupy available transposase binding sites, preventing mobilisation of the *P*-element [8,45,65]. We posited whether the emergence of non-autonomous *P*-element insertions, similar to the *KP*-element, could be responsible for the plateauing of *P*-element copy numbers in the absence of a piRNA-based host defence. To test this, we investigated the coverage and abundance of *P*-element IDs in each replicate over time (Fig 3A). IDs of the *P*-element were detected using DeviaTE, which is based on split-reads (Fig 3A). In both R1 and R3, *P*-element coverage broadly increased throughout the experiment. Although several IDs emerged (black arcs), a contiguous coverage across the entire *P*-element can be observed in R1 and R3, suggesting that abundant full-length insertions are present in these replicates (Fig 3A). In contrast, coverage in R2 increased far more slowly. Several IDs in central regions emerged in R2 at early generations. By generation 63, central regions (positions 1000-1500), are almost completely devoid of coverage, suggesting that extremely few full-length insertions are present in R2 by this time (Fig 3A). To substantiate this, we sequenced 11-12 individual flies from each replicate at generation 98 and estimated *P*-element copy numbers and IDs as described (Fig 3B). Within replicates, *P*-element copies are fairly homogeneous (Fig 3B). Furthermore, the individual flies from R2 have significantly lower copy numbers than those from R1 and R3, consistent with our pooled estimates (Fig 1C). To determine whether full-length insertions are entirely absent in individuals, we next investigated the coverage at an individual level. We reasoned that if a region in the *P*-element has a coverage of zero, then the sample cannot contain a single full-length insertion (Fig 3C, yellow regions). This approach is likely conservative, as non-overlapping IDs in different insertions could result in a contiguous coverage, despite the absence of full-length insertions in the sample. Analysis of individual coverage revealed that at least 6 of the 11 sequenced individuals from R2 lack full-length insertions, whereas we did not find a single sample without full-length in both R1 and R3 (Fig 3C and S8 Fig). Next, we investigated whether any *P*-element insertions with IDs could yield repressors of *P*-element activity, such as the *KP*-element [8]. Such repressors are characterised by two properties: i) transposase translation must be interrupted (due to deletions or premature stop codons) and ii) the DNA-binding domain, in ORF0, must be present (Fig 4A and 4D) [39,45,47]. We aimed to assess the abundance of such putative repressors of *P*-element activity in our experimental populations. We used DeviaTE to identify the breakpoints of the IDs in each population (based on split-reads; Fig 4B) and estimated the frequency of insertions with IDs (based on the number of split reads supporting an ID and the mean coverage). IDs with a frequency <0.05 were filtered out (Fig 4C). We found internally deleted copies that may act as *P*-element repressors in both R2 and R3 (Fig 4C). In R3, we found a single putative repressor at a low frequency (0.05), whereas two with higher frequencies were present in R2 (0.1 & 0.27). Additionally, it is not clear whether another abundant ID (0.24) in R2, in which ORF0 is truncated (potentially reducing DNA binding efficacy), also acts as *P*-element repressor. Hence, putative repressors account for an estimated 0% of the *P*-element insertions in R1, 5% of the insertions in R3 and between 37-61% of the insertions in R2 (Fig 4C). By contrast, full-length insertions, encoding functional transposases, account for 58% of the insertions in R1, 50% of insertions in R3 and only 2% of the insertions in R2. Compared to the other replicates, R2 is characterised by just a few full-length insertions but a high number of copies with IDs that may act as repressors of *P*-element activity. An analysis of the RNA-seq data suggests that the major ID in R1 and the three IDs in R2 are expressed. We did not find evidence that the ID in R3 is expressed (S12 Fig). This further suggests that the IDs in R2 may encode repressors of *P*-element activity. We next asked why *P*-element insertions with IDs rose to a high frequency in our experimental populations, in particular in R2. Three different hypothesis are feasible i) genetic drift ii) preferential mobilisation of insertions with IDs and iii) positive selection of insertions with IDs that repress *P*-element activity. To address this question, we computed a ’fitness landscape’ for IDs along the *P*-element, as described before [39]. The idea being that the frequency of an ID (i.e. the proportion of *P*-element insertions having the ID) reflects its average fitness. For each site in the *P*-element the average frequency of all IDs spanning the site allow us to estimate whether deletion of the site is, on average, favourable or deleterious for attaining a high frequency. In agreement with previous works [29,39], our data suggests that *P*-elements with IDs are preferentially mobilised (S13 Fig). This does not exclude the possibility that these IDs may also act as repressors of *P*-element activity.

We conclude that the plateauing of the *P*-element copy numbers in the absence of a piRNA-based host defence in R2 is likely due to the rapid emergence non-autonomous *P*-elements with IDs. These insertions with IDs are likely preferentially mobilised and additionally have properties similar to known repressors of *P*-element activity.

### Gonadal dysgenesis across experimental populations

The *P*-element was initially discovered as the cause of hybrid dysgenesis (HD) [7,34], where crosses between males having the *P*-element (P strain) with naïve females (M strain) displayed a wide range of different phenotypes, including atrophied ovaries, whereas reciprocal crosses (P females and M males) do not. This non-reciprocity is due to piRNA-based host defences being maternally transmitted, while the *P*-element is transmitted by both parents [11]. Ovary atrophication is a result of germline stem cell arrest due to double strand breaks caused by *P*-element activity [53]. Atrophied ovaries thus provide an easily scored phenotypic indication of *P*-element activity (termed gonadal dysgenesis, GD).

**Fig 5:**
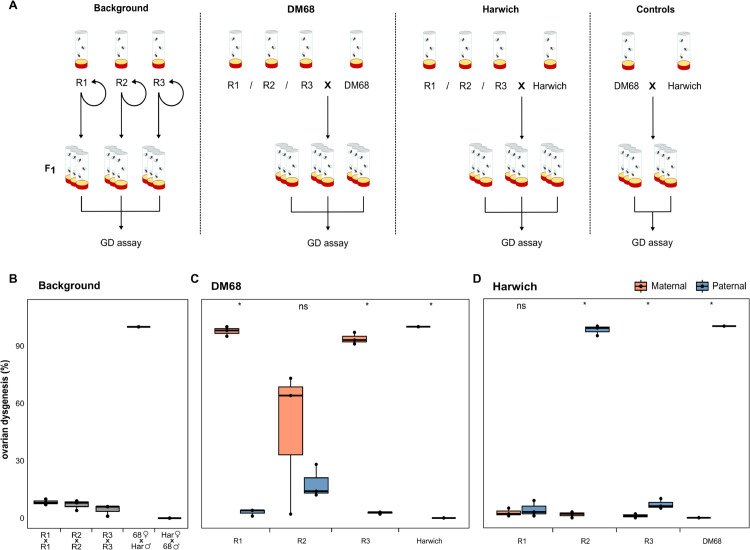
Gonadal dysgenesis (GD) assays suggest that the *P*-element is still weakly active in R2. **A** Schematic overview of crosses. All crosses were performed with flies from the experimental populations at generation 101. **B** Extent of intra-population GD in the experimental populations. As a control, reciprocal crosses among Harwich (with the *P*-element) and DM68 (without the *P*-element) are shown. **C** Crosses of males from the experimental populations to M females (DM68) leads to high levels of GD (orange), while reciprocal crosses show minimal GD (blue). **D** Crosses of females of all experimental populations to Harwich males (a strong GD inducer strain with many *P*-element insertions [34]) induces GD in R2 but not in R1 and R3. Asterisks indicate significant differences in GD levels between reciprocal crosses (*p* < 0.05; paired t-test).

We wanted to ascertain if GD, the hallmark of *P*-element activity, could be detected in our experimental populations. This enables us to test whether *P*-element activity has been reduced by mechanisms other than piRNAs in R2. We performed GD assays at 29°C, i.e. the temperature where *P*-element induced GD is most pronounced [34,35]. For each cross, we used three sub-replicates of four males and four females, and then dissected ovaries of the F1. Crosses between the strong P strain, Harwich [34], and the M strain, DM68 (S1 Fig), acted as controls (Fig 5A). We expect strong GD for crosses between Harwich males and DM68 females, but no GD in the reciprocal crosses. From the intra-population GD assays, we detected little GD across all sub-replicates, indicating a low level of activity in all experimental populations (Fig 5B), further highlighting that *P*-element activity is low within the R2 population. Interestingly, levels of intra-population GD were consistently low throughout the experiment for R2, whereas GD-levels were initially low (until generation 15) for R1 and R3, but rose to ∼80% by generation 25, then dropped again to lower levels at generation 40 (S9 Fig).

Next, we tested if *P*-element insertions in the experimental populations are able to induce GD, informing us as to whether they are still functional. We crossed males from the experimental replicates to the females of DM68 (M strain). As DM68 contains no *P*-element insertions and therefore no complementary piRNAs, their offspring will exhibit GD if males have sufficient numbers of functional *P*-element insertions. We found that crosses of males from all replicates with DM68 females induced GD, while reciprocal crosses did not (Fig 5C). Interestingly, we see that the *P*-element in R2 could still induce a substantial amount of GD, albeit at a lower and more variable level than seen in R1 and R3. However, in R2 the reciprocal cross (R2 females with DM68 males) also has a slightly elevated GD level.

Lastly, we tested if the different replicate populations are able to silence the *P*-element, by crossing experimental females to Harwich. If females from the experimental populations have *P*-element piRNAs, we should expect little to no GD. Crosses with females from R1 and R3 did not exhibit GD consistent with the emergence of a piRNA-based host defence (Figs 1D, 2 and 5D). In contrast, crosses with females from R2 show strong GD (Fig 5D), indicating that a piRNA-based host defence against the *P*-element is still absent in R2 after over 100 generations of the experiment.

Taken together, our GD assays suggest that an effective host defence (likely piRNAs) emerged in both R1 and R3, but not in R2. Nevertheless, R2 still contains functional *P*-element copies, which are able to induce GD. The absence of intra-population GD in R2 further suggests that the *P*-element activity is low in this replicate.

### Internally deleted *P*-elements seen in global populations

**Fig 6:**
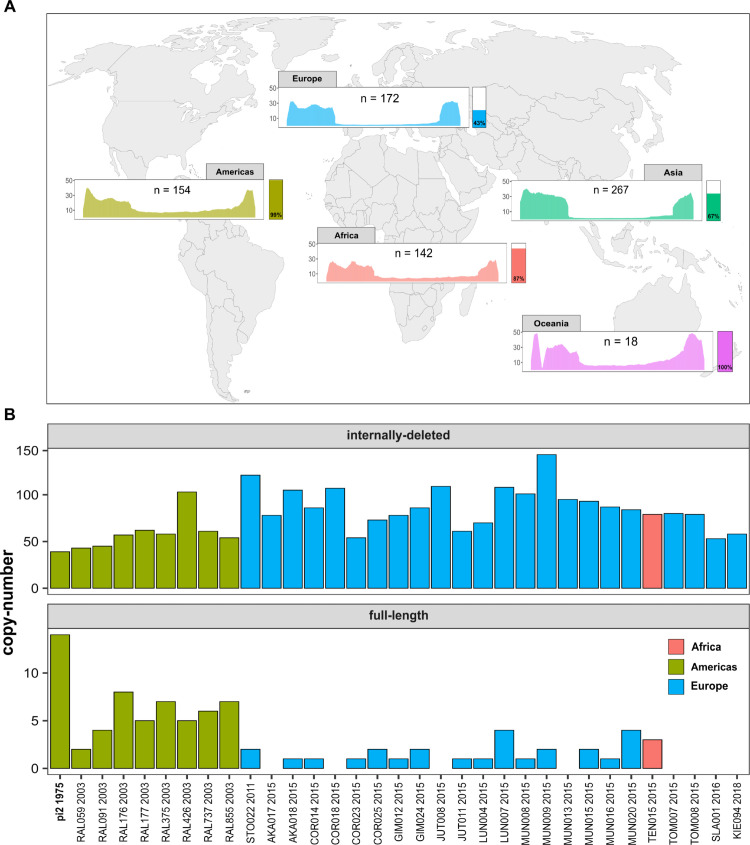
Abundance and structure of *P*-element insertions across natural *D. melanogaster* populations. **A** Mean coverage of the *P*-element in different geographic regions (data from 753 short-read datasets). Bars on the right provide a rough estimate of the fraction of samples containing at least a single full-length insertion. **B** Copy number of full-length *P*-element insertions and of insertions with IDs in long-read assemblies of recently collected *D. melanogaster* strains [66]. Strains are coloured by region and labels show strain name and collection year. pi2 (bold) is a frequently used inducer strain of GD, collected in 1975. World map SVG (https://commons.wikimedia.org/wiki/File:BlankMap-World.svg).

We sought to assess whether an absence of full-length insertions in individuals, as seen in R2 (Fig 3), could also be observed in natural *D. melanogaster* populations. Previous works raise the possibility that individuals with ID elements but no full-length insertions might be found in some natural populations [28,56,80]. To substantiate these findings we performed a survey of *P*-element composition in worldwide populations using publicly available data. Initially, we utilised a total of 753 short-read samples (strains or pooled populations) collected from all major continents [14,23,30,40,61,66,73]. The average normalised *P*-element coverage across different continents shows that coverage frequently decreases within central regions of the *P*-element (Fig 6A). The coverage dip is likely due to highly abundant IDs, such as the *KP*-element [8]. This is in agreement with previous works, reporting abundant full-length insertions in North America and many ID elements in populations from Europe and Africa [3,4,8,28,86]. Based on this, we estimate that the fraction of samples containing at least one full-length insertion varies dramatically across continents. Our data suggests that full-length insertions of the *P*-element are rare in both Europe and Asia but more abundant in the Americas, Africa, and Oceania (Fig 6A). However, this data needs to be treated with some caution, as we only considered the coverage in central regions of the *P*-element and as we have included pooled populations in our analysis.

To further investigate the composition of the *P*-element in natural populations, we analysed 33 long-read assemblies of *D. melanogaster* strains, recently collected from Europe and North America [13,26,66,88]. We used RepeatMasker to identify full-length insertions and insertions with IDs in these assemblies. All investigated strains contained *P*-element insertions (either full-length or insertions with IDs; Fig 6B). In agreement with the short-read data, we found that full-length insertions were rare in strains from Europe but more abundant in strains from North America (Fig 6A). Several of the European strains collected between 2015 and 2018 did not contain even a single full-length *P*-element insertion (Fig 6B).

Our data shows that the absence of full-length insertions in populations invaded by the *P*-element, as observed in our R2, may also occur in natural *D. melanogaster* populations.

## Discussion

We introduced the *P*-element into three replicate populations of *D. melanogaster* and monitored the following invasion at the level of the genome and transcriptome for over 100 generations. We observed that copy numbers of the *P*-element stabilised at around 20-25 copies in two replicates (R1, R3), but at only ∼7 copies, in R2. Interestingly, copy numbers stabilised in R2 despite the absence of a piRNA-based host defence (until at least generation 45, from small RNA data). GD assays indicate that a piRNA-based host defence was still absent in R2 at generation 98 (females crossed with Harwich males induced GD; Fig 5D). We found that non-autonomous *P*-element insertions with IDs rapidly emerged and proliferated in R2. Many individuals from R2 in later generations contain *P*-element insertions with IDs but are without a single full-length insertion. Several of these IDs share features of repressors of *P*-element activity, similar to the *KP*-element. We posit that the early appearance and propagation of non-autonomous *P*-element insertions is responsible for the stabilisation of *P*-element copy numbers in R2, despite the absence of a piRNA-based host defence. At generation 98, 6 of 11 individuals sampled from R2 did not contain a single full-length insertion (Figs 3 and S8 Fig). We found that individuals without full-length insertions were also observed in natural populations (Fig 6). This does however not imply that the evolutionary dynamics shaping natural and experimental populations were identical. It is likely that the *P*-element composition in natural populations were shaped by multiple migration events combined with the emergence of novel IDs, whereas migration was likely not an important factor in our experimental populations [3,63,86].

It has been suggested in previous works that non-autonomous elements may outcompete full-length insertions [39,62,68]. However, our work shows for the first time that non-autonomous insertions may emerge *de novo* within but a few generations in experimental populations and then proliferate to such an extent that TE copy numbers stabilise despite the absence of the host defence. We suggest that this proliferation was driven by preferential mobilisation of *P*-element insertions with IDs. This is in agreement with previous works suggesting that *P*-element insertions with IDs are likely preferentially mobilised [29,38,39,54,74]. A mobilisation advantage of non-autonomous elements has also been noted for other TE families [27]; in a direct competition, non-autonomous *Mariner* insertions were able to outcompete their autonomous counterparts [68]. It is feasible that the shorter length of non-autonomous insertions facilitates a more effortless transposition [54]. Our work also highlights a novel risk for TEs, i.e. that TE invasions can fail due to the emergence of non-autonomous elements. Furthermore, our work highlights that TE invasions may reach stable copy numbers in the absence of a piRNA based host defence. It remains an important open question as to whether mechanisms other than IDs can also lead to stable TE copy numbers in the absence of piRNAs, as observed in [84].

Our work also leads us to consider why the piRNA-based host defence never established itself in R2. It is not yet clear what triggers the emergence of a piRNA-based host defence, but insertions in piRNA clusters, or siRNAs mediating the conversion of TE insertions into piRNA producing loci, have been suggested as potential mechanisms [5,10,46]. It is possible that *P*-element copy numbers in R2 were insufficiently abundant to trigger these mechanisms. In a previous study, we described another replicate population of *D. erecta*, wherein the host defence against an invading *P*-element also failed to be established [74]. Copy numbers of the *P*-element in *D. erecta* were over an order of magnitude higher than in this work (*D*.*ere* = 151, *D*.*mel* = 7) and acted to the severe detriment of the population’s fitness. This suggests that the mechanism triggering the host defence in *Drosophila* may not depend on TE copy numbers, as in plants [49]. In *D. erecta*, we also found multiple *P*-element insertions in piRNA clusters and a significant number of *P*-element siRNAs, suggesting that these two mechanisms are insufficient to trigger the establishment of the host defence [74]. Interestingly, non-autonomous *P*-element insertions with properties similar to the *KP*-element also proliferated in the unprotected *D. erecta* population [74]. This indicates that abundant *P*-element insertions with IDs might interfere with the establishment of a piRNA-based host defence. The timing of the ID emergence within the population is likely critical, as we observe a prominent ID appearing early on in R2 (Fig 3A). A later emergence of IDs may not be sufficient to stop an invasion without activating the piRNA-based host defence system. The mechanism by which this could be achieved remains unclear.

Another open question is why R2 males induced GD when crossed with DM68 females (M strain) but not with R2 females, despite both lacking a piRNA-based host defence (Fig 5). We do not have a definitive answer to this, but we speculate that it could be linked to the dosage of non-autonomous elements. While the piRNA based host defence is maternally inherited, the repressive effects of the *KP*-element, and other non-autonomous insertions that may affect P-element activity, is transmitted by both parents [8]. Hence, the offspring of crosses of R2 males with R2 females end up with twice as many non-autonomous insertions as the offspring of R2 males with DM68 females and this higher dosage of non-autonomous insertions may be necessary to prevent *P*-element mobilisation. However, it must be noted that previous studies found no correlation between the *P*-element composition (i.e. abundance of full-length insertions and fraction of ID elements) [4,28]. Only [81] showed that the proliferation of very short *P*-element insertions (Har-P) could be responsible for the high rate of GD induced by some strains, such as Harwich. It is therefore an open question as to which genomic factors influence the extent of GD. It is perhaps worth noting that the flies from R2 closely resemble the rare P’ strains [31]. These strains are able to induce GD (when crossed paternally to an M strain), yet at the same time are susceptible to GD (when crossed maternally to a P strain) [31]. This implies that P’ strains have active *P*-element insertions but no maternally transmitted piRNAs. One explanation is that the P’ phenotype is a transient stage that can only be observed for a few generations in strains actively being invaded by the *P*-element. Our findings raise the possibility that for some strains the P’ phenotype may be stable, wherein *P*-element activity might be controlled by non-autonomous insertions, *in lieu* of a piRNA-based host defence.

This study challenges our understanding of the evolutionary impact of failed invasions, where non-autonomous elements have proliferated at the cost of full-length insertions. Such failed invasions could pose a severe threat to the long-term sustained survival of TEs. TEs silenced by a host are likely to accumulate mutations over time, eventually resulting in the loss of functional copies within the host population [9,72]. To persist, TEs must invade novel species, e.g. following horizontal transfer (HT). HT is likely a rare event, and TEs that fail to take advantage of the limited opportunities to spread into novel species may be unable to persist. The rapid proliferation of non-autonomous insertions, as observed in R2, poses a two-fold threat to the prolonged existence of TEs. First, abundant IDs could effectively ’immunise’ a species (or population) to further invasions from a TE. Any newly introduced full-length insertions (e.g. recurrent HT) may be quickly outcompeted by the non-autonomous insertions already pervasive in the species. Second, species with abundant non-autonomous elements are likely not ’infective’. Due to the scarcity of full-length insertions, HT from populations with abundant non-autonomous insertions to M species is unlikely to trigger a TE invasion. Populations with abundant non-autonomous insertions are likely evolutionary dead-ends: resistant to further invasions, yet unable to infect other species. Consistent with previous works, we show that a proliferation of non-autonomous *P*-element insertions can also be observed in natural populations of *D. melanogaster*, where especially those from Europe have few full-length insertions (Fig 6 [3,4,8,28,86]). These European populations could exhibit the two-fold cost of the proliferation of non-autonomous elements, threatening the long-term persistence of TEs.

## Materials and methods

### Experimental populations

We introduced the *P*-element into DM68, a *D. melanogaster* strain collected 1954 in Israel, via micro-injection of the plasmid ppi25.1 (kindly provided by Dr. Erin Kelleher). Injections were performed by Rainbow Transgenic Flies Inc (https://www.rainbowgene.com/; Camarillo, CA, USA). We obtained 7 lines containing the *P*-element by crossing transformed adults (2 males and 3 females). Transformed lines were maintained at 20° C for 3 generations before setting up the experimental populations.

To establish the experimental populations, we crossed five males from five *P*-element containing lines with five DM68 (M strain) virgin females and allowed them to mate for 3 days. After mating, we mixed these 50 flies [(5M+5F)*5] from the crosses with 200 DM68 *D. melanogaster* flies. We maintained 3 replicates of the experimental populations with a population size of *N* = 250 for over 100 generations at 25°C using non-overlapping generations.

### Genomic analysis

For genomic sequencing, we sequenced pools of 60 flies using Illumina 2 × 125bp reads. The individual flies at generation 98 were sequenced by BGI with 150bp reads (BGI Tech Solutions, Hong Kong). The abundance of the *P*-element was estimated with DeviaTE. Illumina short reads were aligned to a list of the consensus TE sequences in *D. melanogaster* (https://github.com/bergmanlab/drosophila-transposons [64]), alongside the *D. melanogaster* reference genome (r6.51), including three single-copy genes; *tj*, *RpL32* and *rhi* (FlyBase release 2017_05). Coverage of the TEs was normalised to the abundance of the single-copy genes to estimate the abundance of the TE. DeviaTE was also used to obtain information about strucural variants within the *P*-element.

### Transcriptomic analysis

RNA was collected and sequenced from 30 female flies, either from whole-fly tissue or ovaries. Small RNA and RNA from these samples was sequenced by Fasteris (https://www.fasteris.com/en-us/) and BGI (BGI Tech Solutions, Hong Kong). RNA samples were treated with DNase and poly-A selected before they were sequenced using Illumina 2 × 100bp reads (NovoSeq). RNA data were aligned using GSNAP (version 2014-10-22; [89]) to the reference of *D. melanogaster* (r6.52; Flybase) combined with the consensus sequences of TEs in *D. melanogaster* ([4]). The coverage and the splicing level of the *P*-element were visualised in R. Adaptor sequences of the small RNA data were removed with cutadapt (v2.6 (Martin, 2011)). We aligned the small RNA data to the *D. melanogaster* transcriptome (r6.62, Flybase) combined with the consensus sequences of TEs using novoalign (v3.09.00; http://www.novocraft.com/). The abundance of piRNAs, the distribution of piRNAs within the *P*-element, the length distribution of the piRNAs, the ping-pong and phasing signature were computed using previously described Python scripts [39,74].

### Properties of *P*-element insertions with IDs

We investigated the most abundant IDs in all replicate populations using the combined data from individual flies sequenced at generation 98. ID positions were inferred from split-reads, aligned by DeviaTE (see above). Frameshifts and premature stop codons were identified using ORFfinder [69] and Expasy [17]. We estimated the frequency of the IDs based on the count of split-reads relative to the coverage. First for each replicate we calculated the average coverage of *P*-element regions not covered by IDs, excluding 50 bp at either end to avoid lower coverage regions. Next, we computed the proportion of each individual ID in the populations as the count of split-reads divided by the mean coverage outside of regions with IDs. Frequencies of the full-length germline mRNA were estimated as the minimum coverage of the *P*-element across all individuals of a replicate, again divided by the mean coverage outside of regions with IDs.

### Gonadal dysgenesis

Gonadal dysgenesis assays were set-up using 3 sub-replicates, with the exception of the intra-population assays conducted during the experiment where a single replicate was used (S9 Fig). To estimate the level of GD for each cross we allowed 4 virgin females and 4 males to mate for 2 days. Selected flies were placed in cages and left to lay eggs for 2 days. The remaining eggs were kept at a constant 29°C until flies eclosed. The females were then taken for dissection. For each sub-replicate, we dissected 50 flies (100 ovaries) in 1x PBS solution and scored the proportion of atrophied ovaries.

### *P*-element in natural populations

We gathered a total of 753 publicly available short-read datasets ([14,23,30,40,61,66,73]) and 33 long-read assemblies ([13,26,66,88]). For the short-read data, we estimated the abundance of the *P*-element with DeviaTE, as described above. We assumed that samples with a normalised coverage >1 over the whole sequence have at least one full-length insertion. To analyse *P*-element composition in the long-read assemblies, we used RepeatMasker [78] (open-4.0.7; -no-is -s -nolow) with a custom library that included only the *P*-element consensus sequence. Samples with at least one insertion with a length >2325 bp (80% of the *P*-element with 2907 bp) are considered to contain a full-length insertion.

## Supporting information

S1 Fig IGV coverage of the *P*-element and the single-copy gene (SCG) RpL32 in the M strain, DM68. (TIF)

S2 Fig siRNAs and piRNAs mapping to the *P*-element in all experimental populations across time. (TIF)

S3 Fig Length distribution of the small RNAs mapping to the *P*-element for each replicate. (TIF)

S4 Fig Distribution of mapped piRNAs (23-29nt) across the *P*-element during the experiment. (TIF)

S5 Fig Ping-pong signatures for the *P*-element for all replicates. (TIF)

S6 Fig Ping-pong signatures for *Blood* (a LTR retrotransposon) for all three replicates. (TIF)

S7 Fig Phasing signatures for the *P*-element for all replicates. (TIF)

S8 Fig DeviaTE coverage plots for 11-12 individual flies of each replicate at generation 98. (TIF)

S9 Fig The extent of intra-population gonadal dysgenesis throughout the experiment. (TIF)

S10 Fig *P*-element expression for each replicate. (TIF)

S11 Fig Sense and antisense expression of the *P*-element during the experiment. (TIF)

S12 Fig mRNA expression of high frequency IDs in all replicates. (TIF)

S13 Fig Normalised frequency of internal deletions. (TIF)

S14 Fig Estimated insertion sites and their population frequencies for each replicate across time, using PoPoolationTE2. (TIF)

S1 Table Results of all gonadal dysgenesis assays from each listed cross. (XLSX)

S2 Table All identified *P*-element SNPs throughout the invasion. (XLSX)
