# Supplementary figures and images for: Rapid emergence of non-autonomous elements may stop P-element invasions in the absence of a piRNA-based host defence
Source: PLoS Genet. 2025 Aug 20;21(8):e1011649. doi: 10.1371/journal.pgen.1011649 (PMC12393704; doi:10.1371/journal.pgen.1011649)

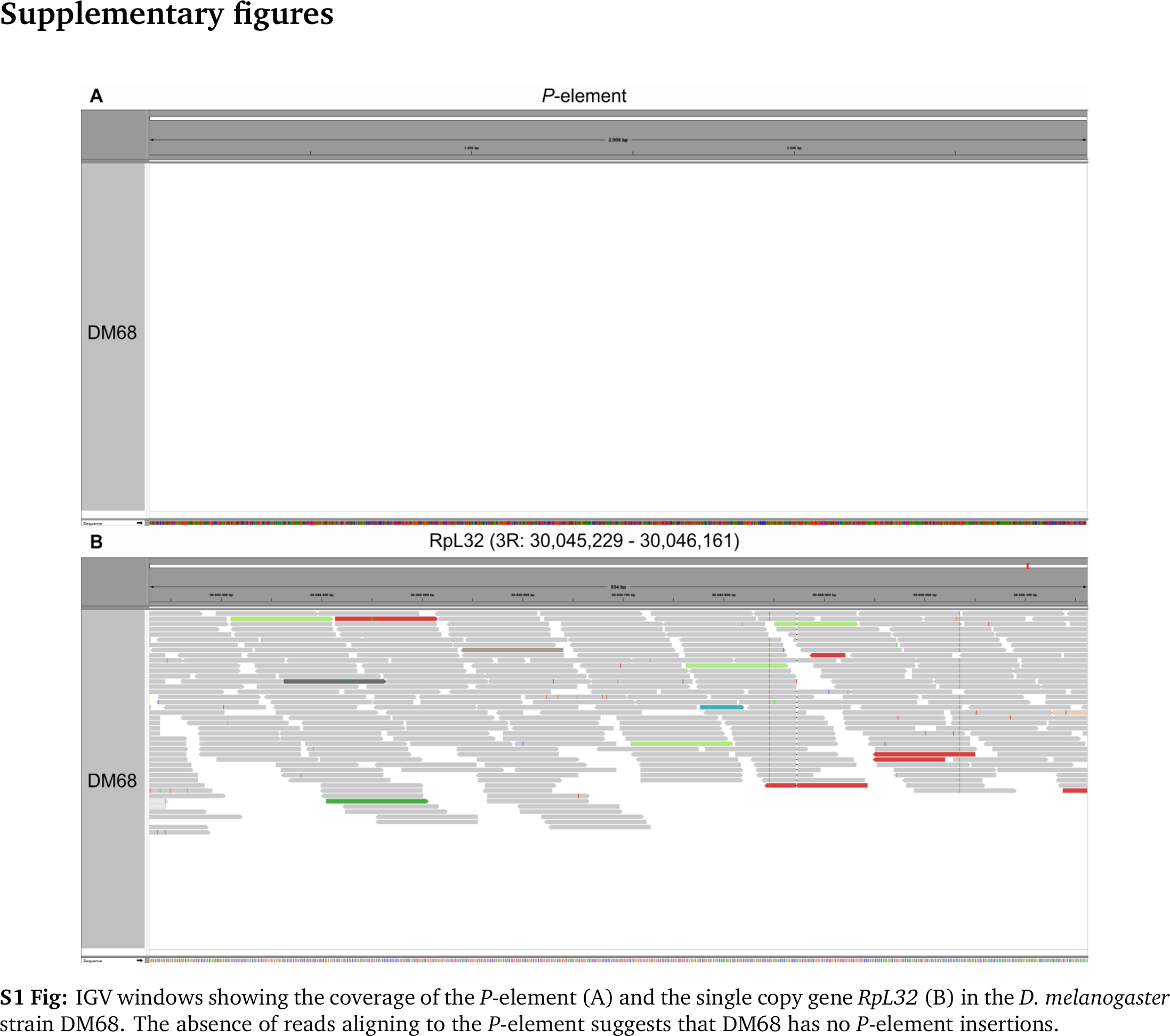

Supplement: S1 Fig — (TIF) [file pgen.1011649.s001.tif]

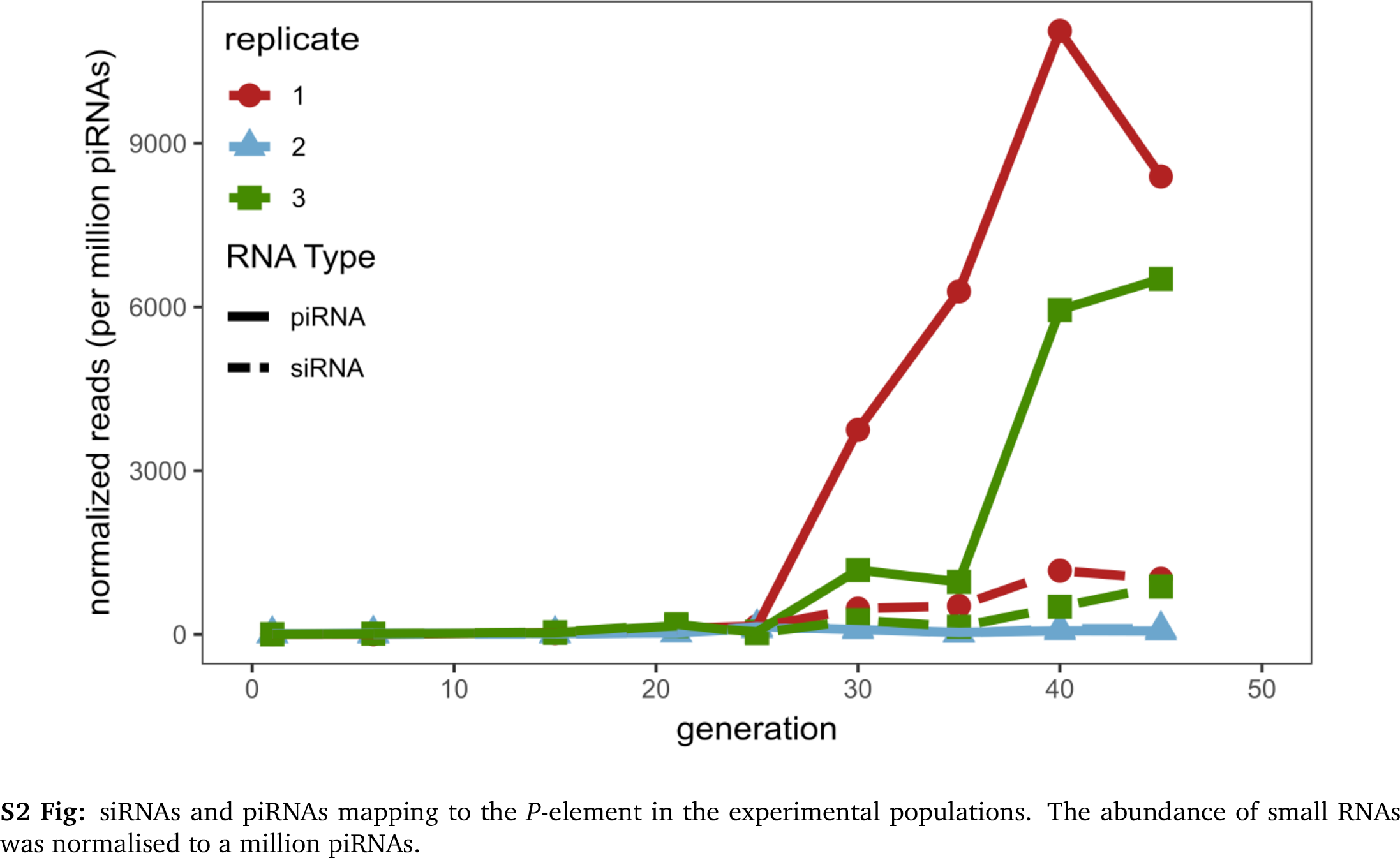

Supplement: S2 Fig — (TIF) [file pgen.1011649.s002.tif]

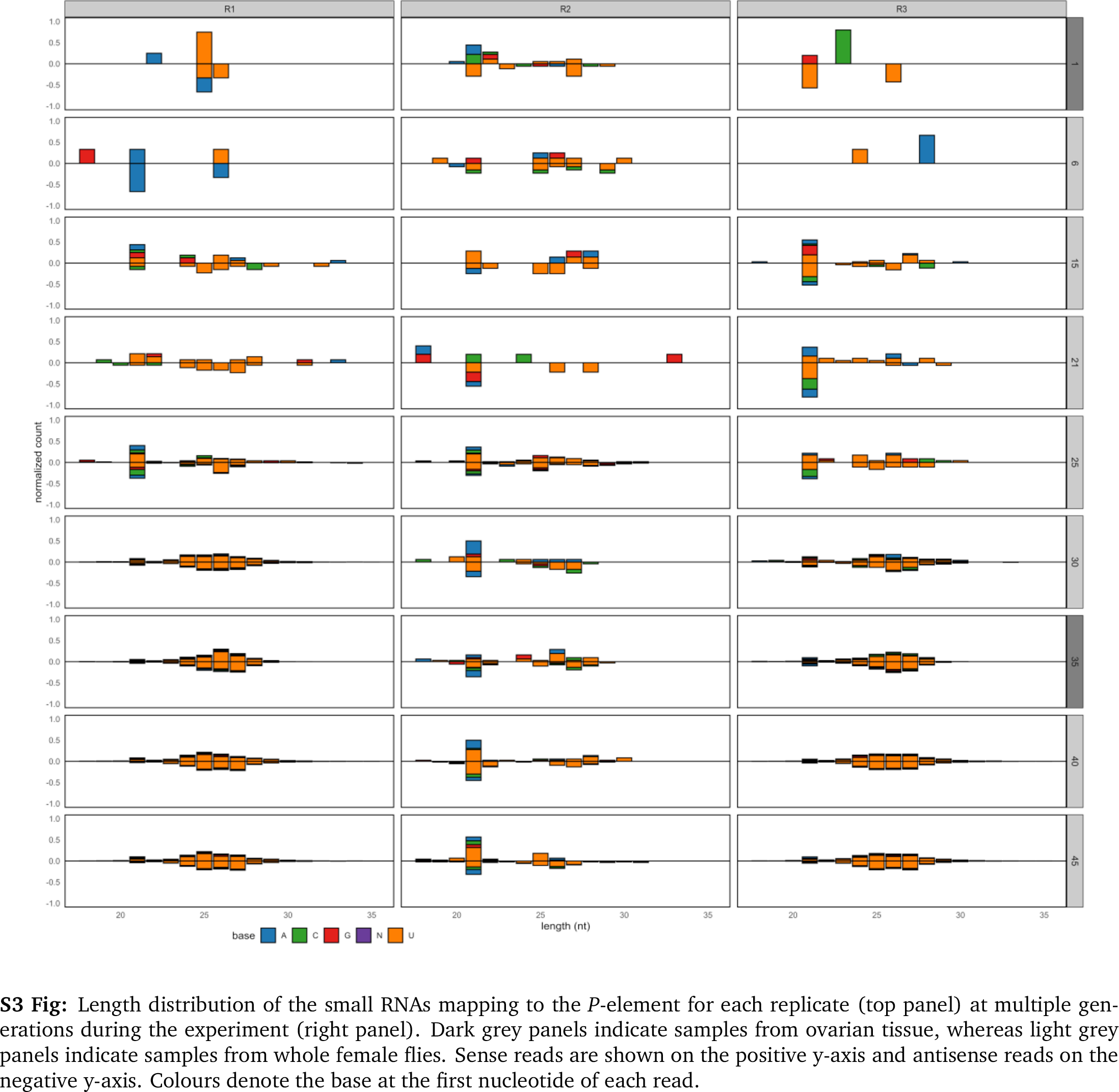

Supplement: S3 Fig — (TIF) [file pgen.1011649.s003.tif]

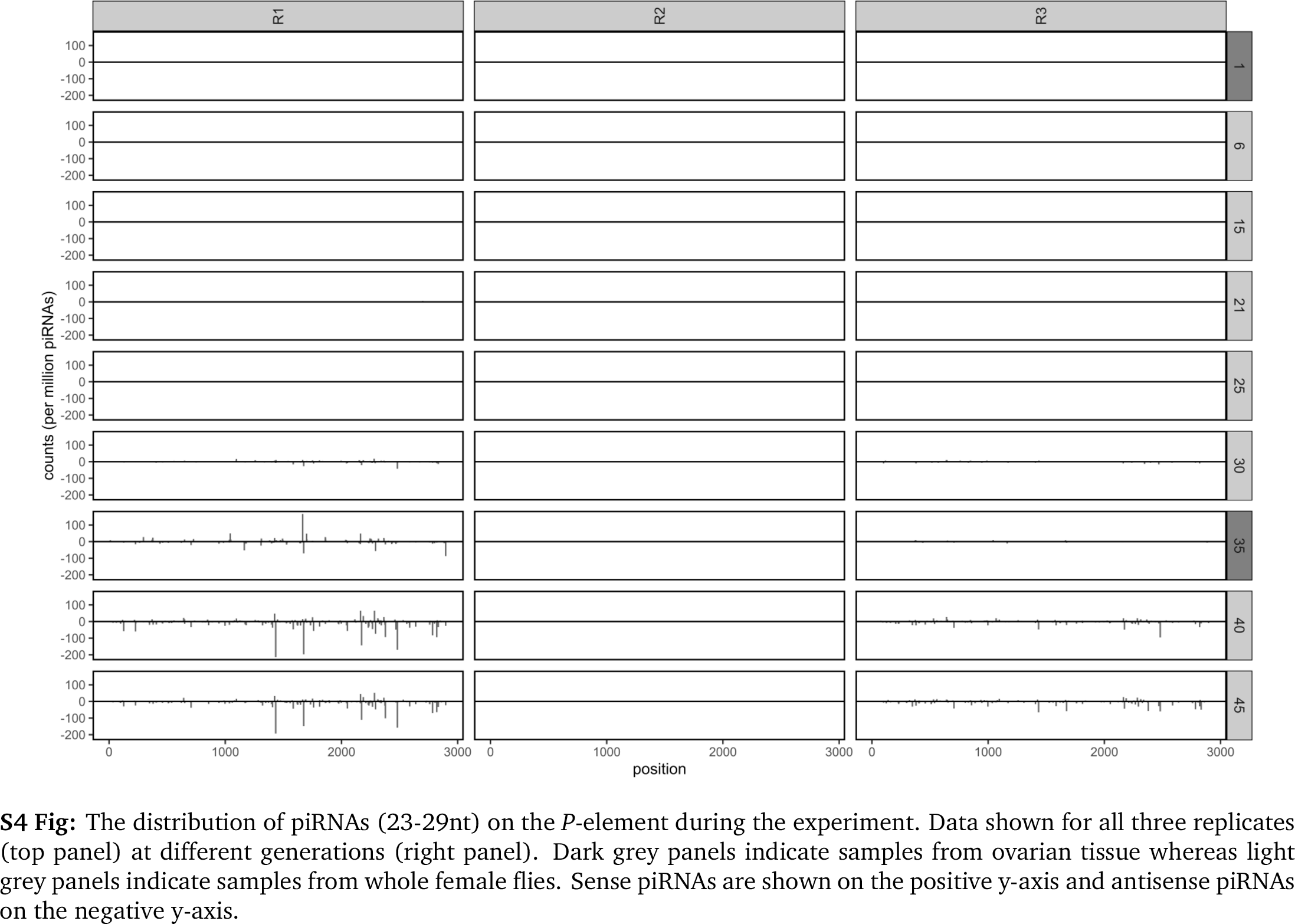

Supplement: S4 Fig — (TIF) [file pgen.1011649.s004.tif]

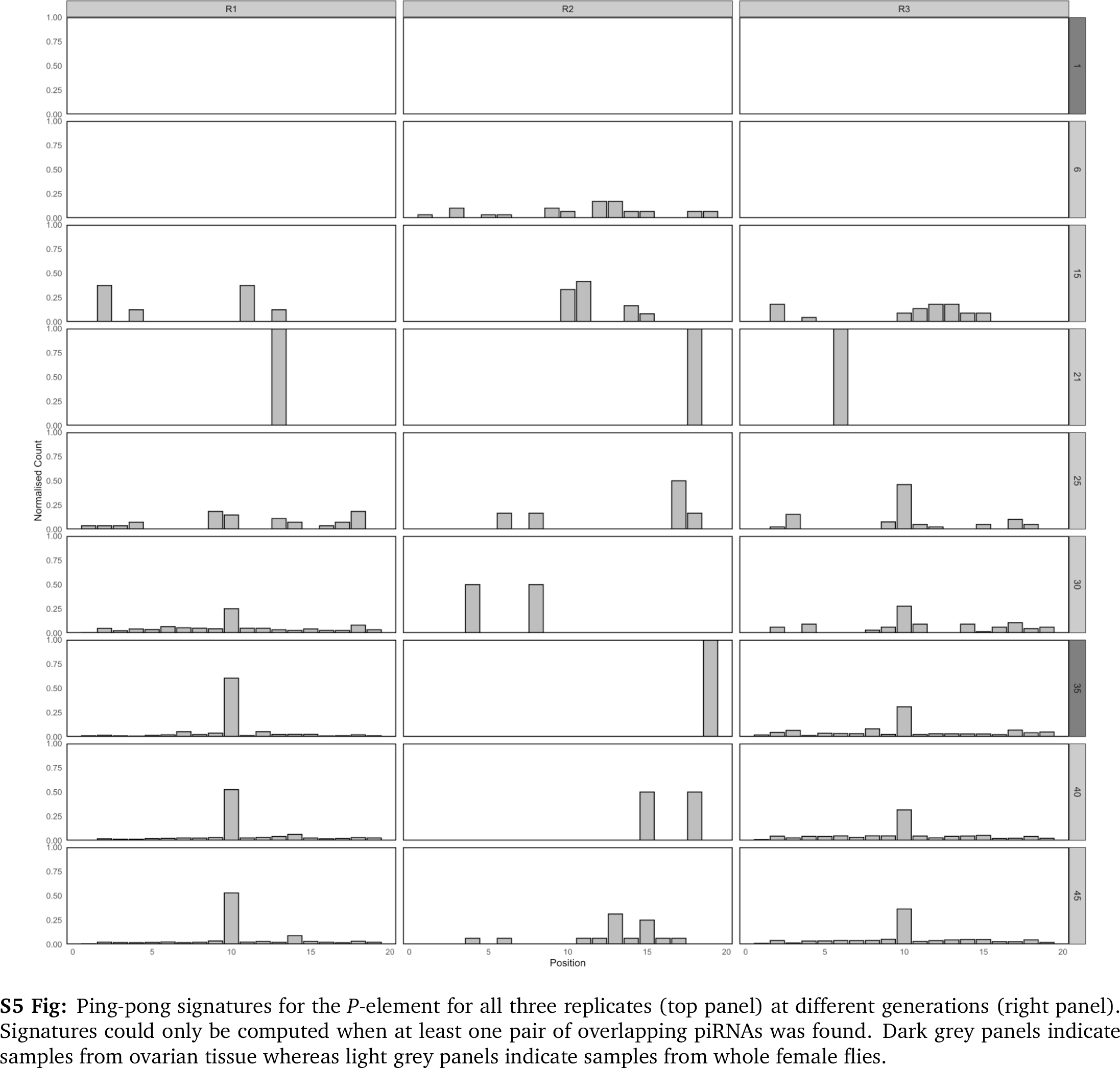

Supplement: S5 Fig — (TIF) [file pgen.1011649.s005.tif]

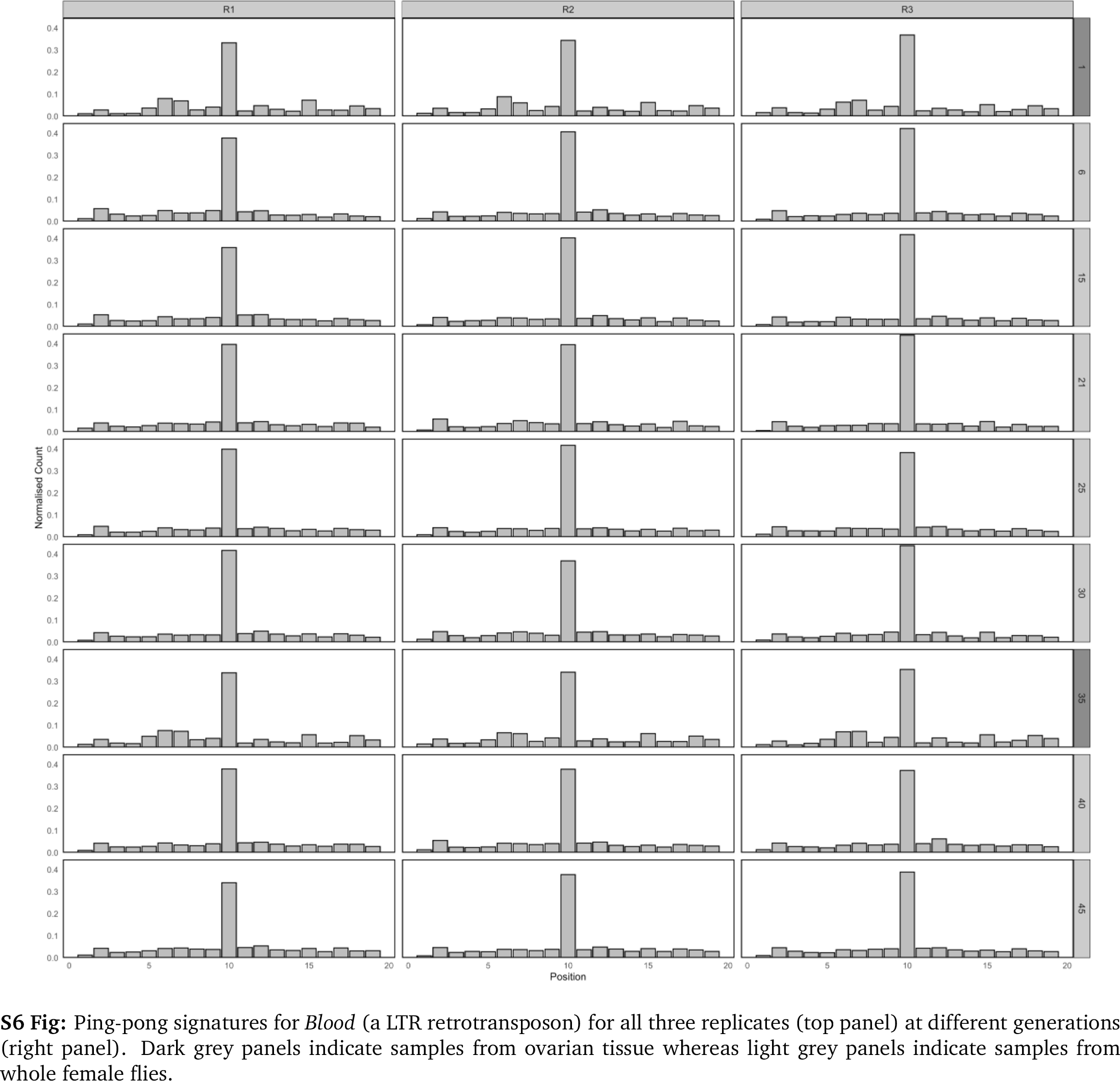

Supplement: S6 Fig — (TIF) [file pgen.1011649.s006.tif]

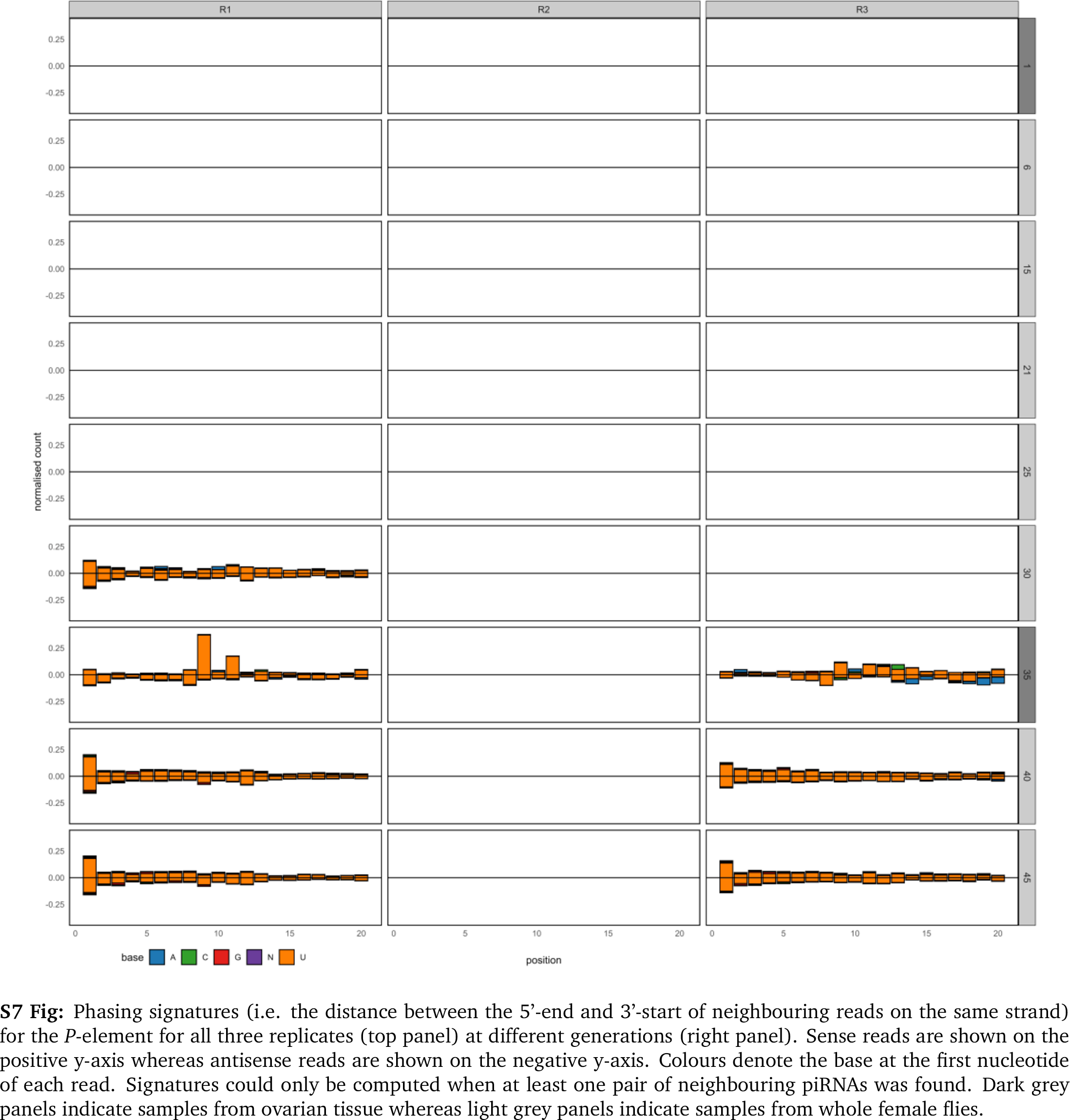

Supplement: S7 Fig — (TIF) [file pgen.1011649.s007.tif]

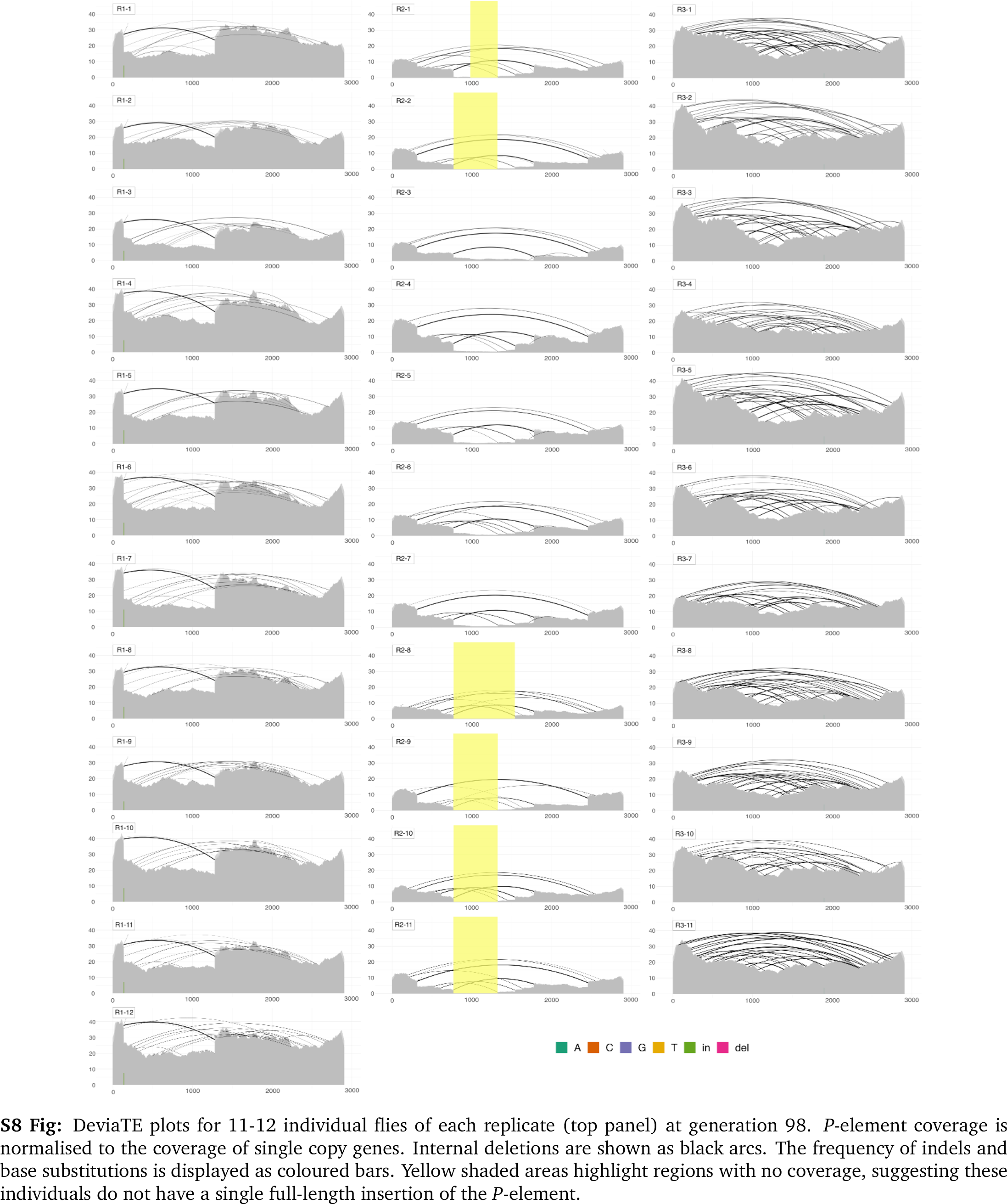

Supplement: S8 Fig — (TIF) [file pgen.1011649.s008.tif]

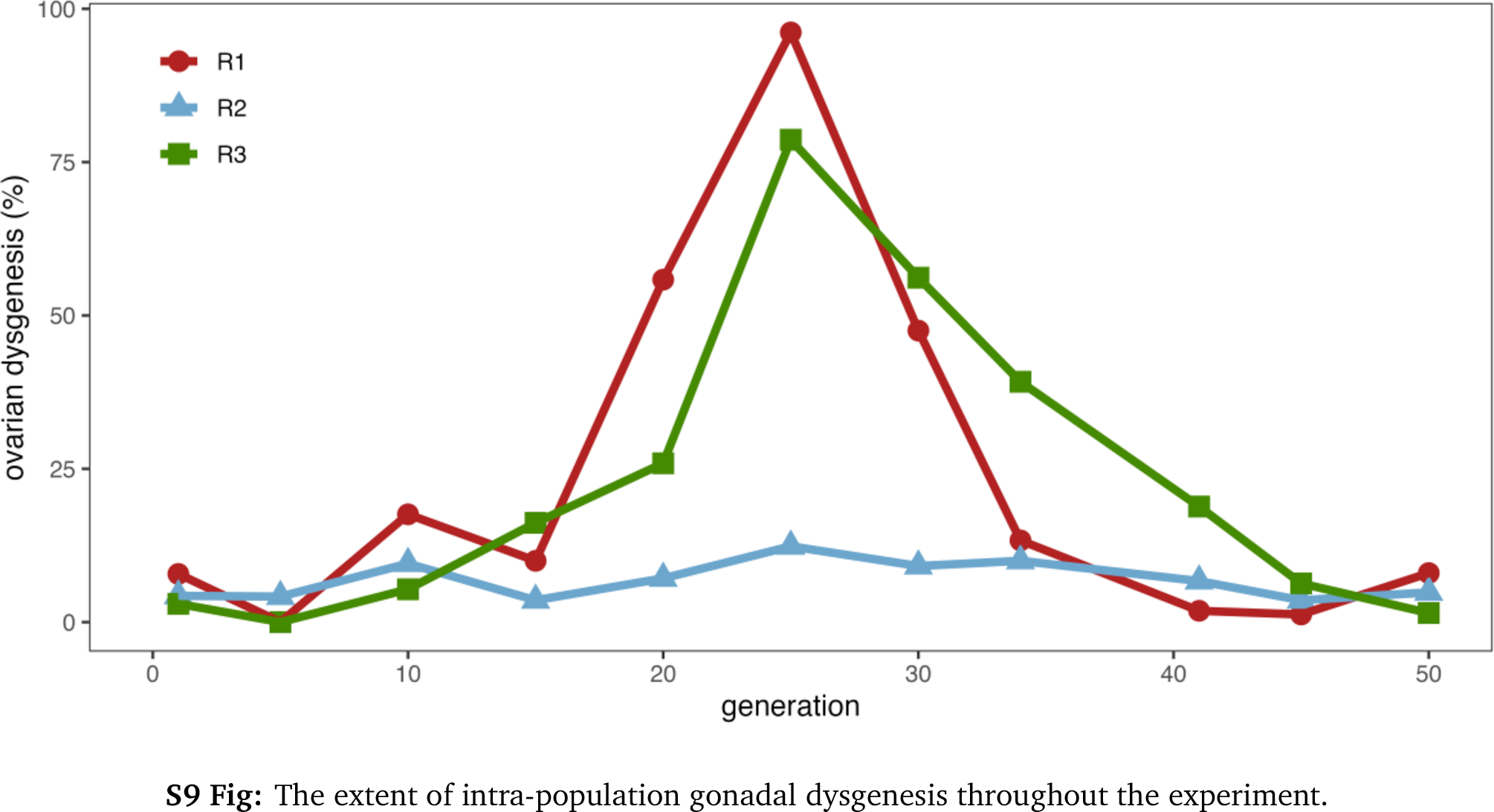

Supplement: S9 Fig — (TIF) [file pgen.1011649.s009.tif]

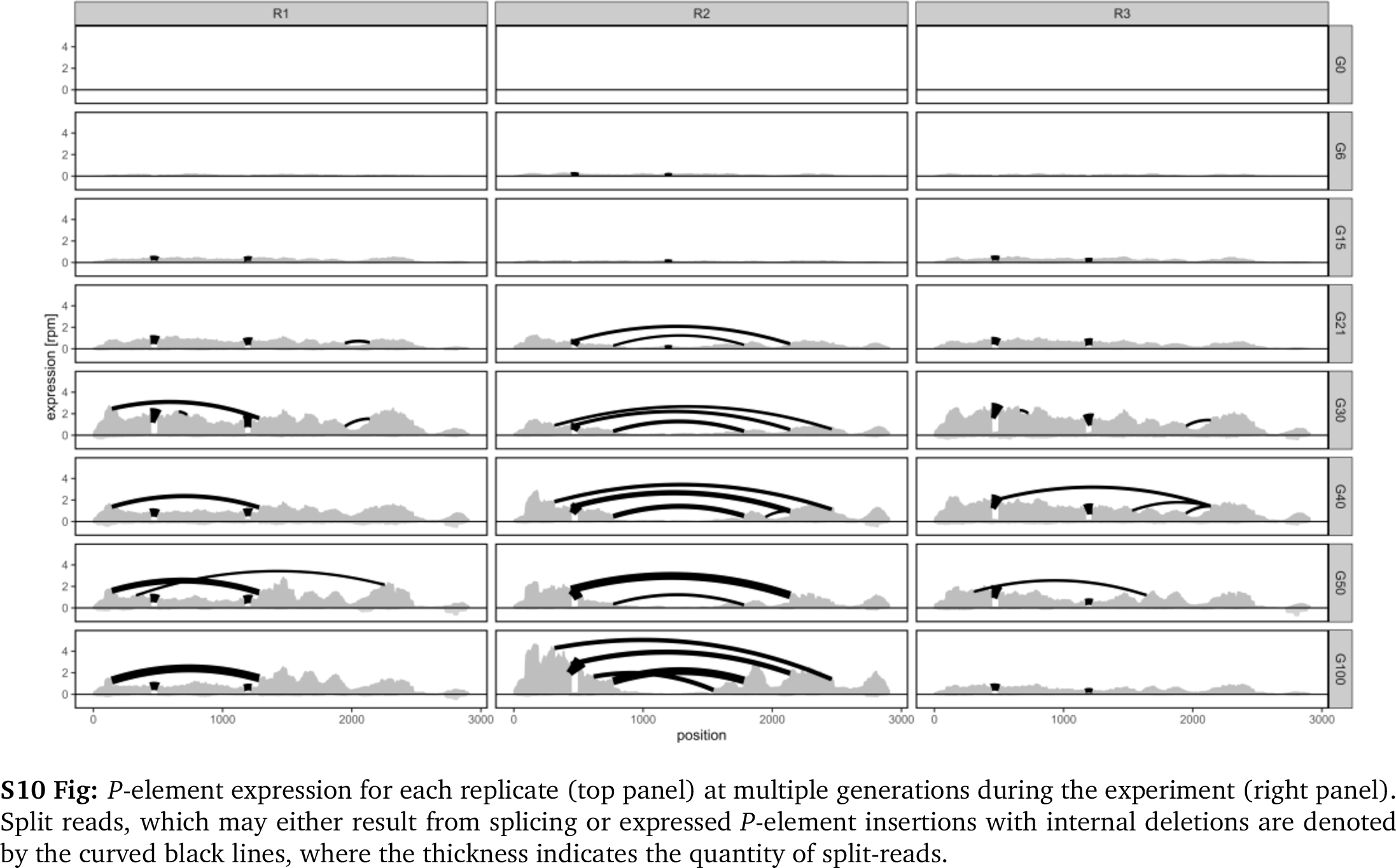

Supplement: S10 Fig — (TIF) [file pgen.1011649.s010.tif]

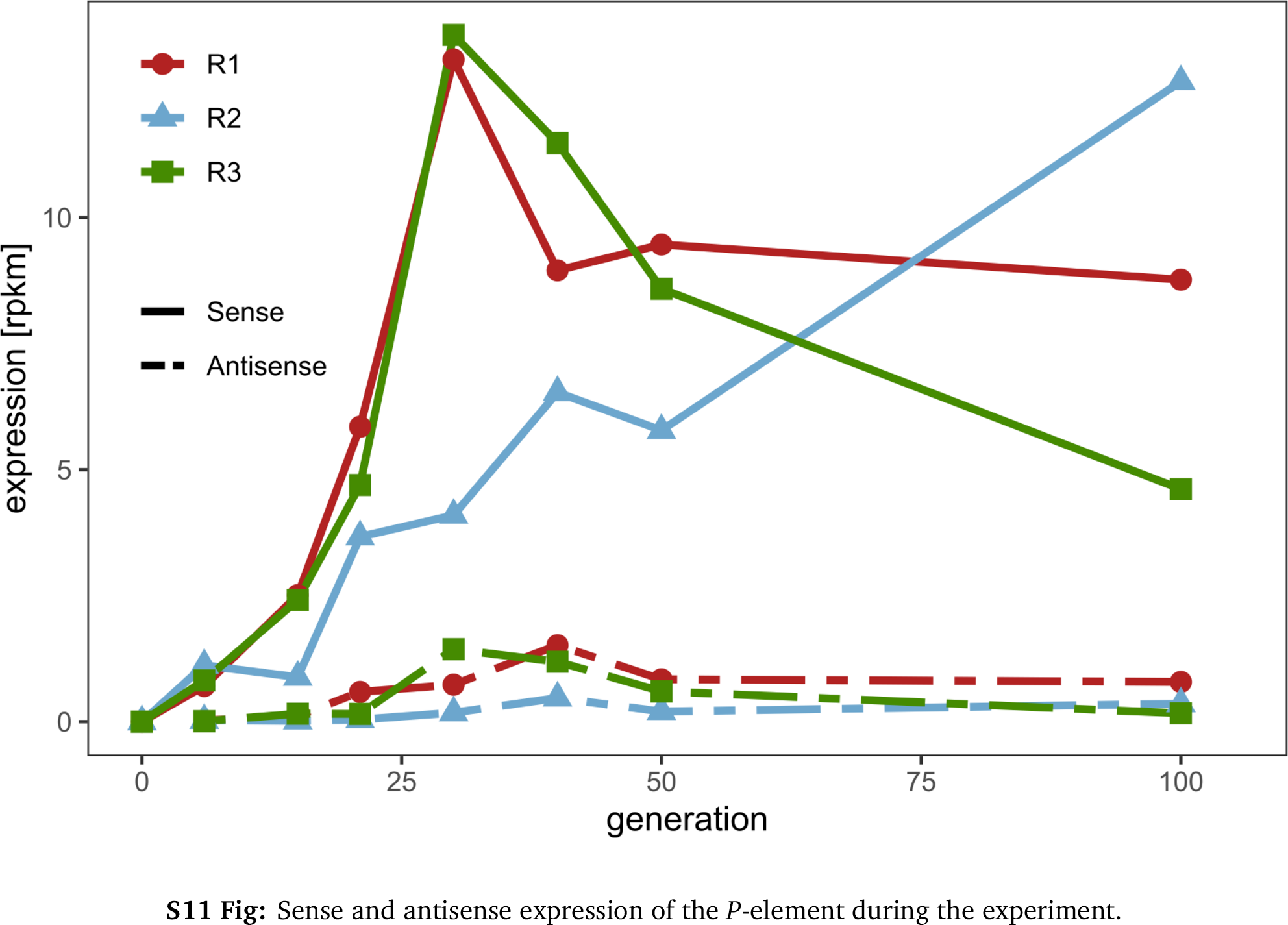

Supplement: S11 Fig — (TIF) [file pgen.1011649.s011.tif]

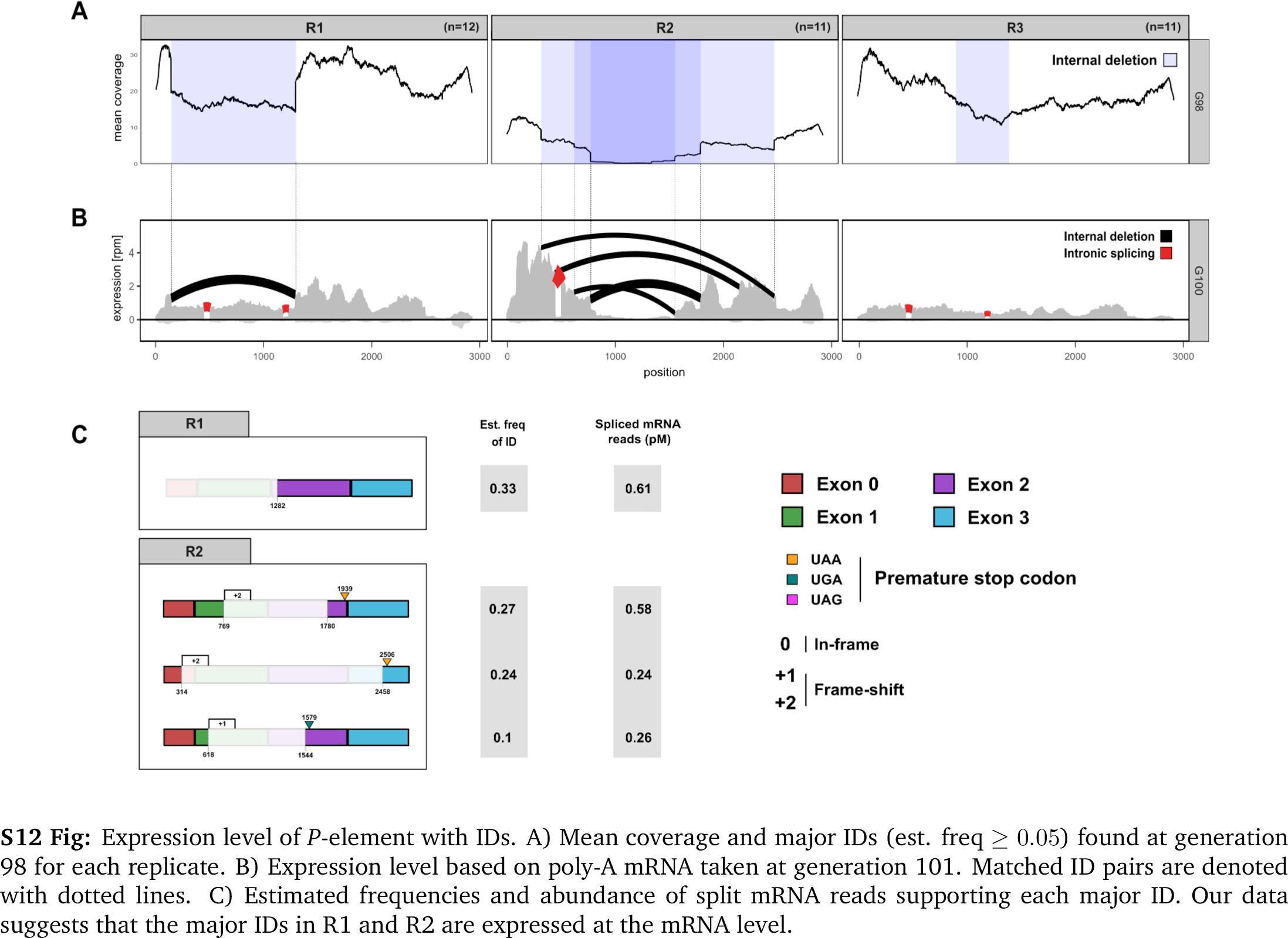

Supplement: S12 Fig — (TIF) [file pgen.1011649.s012.tif]

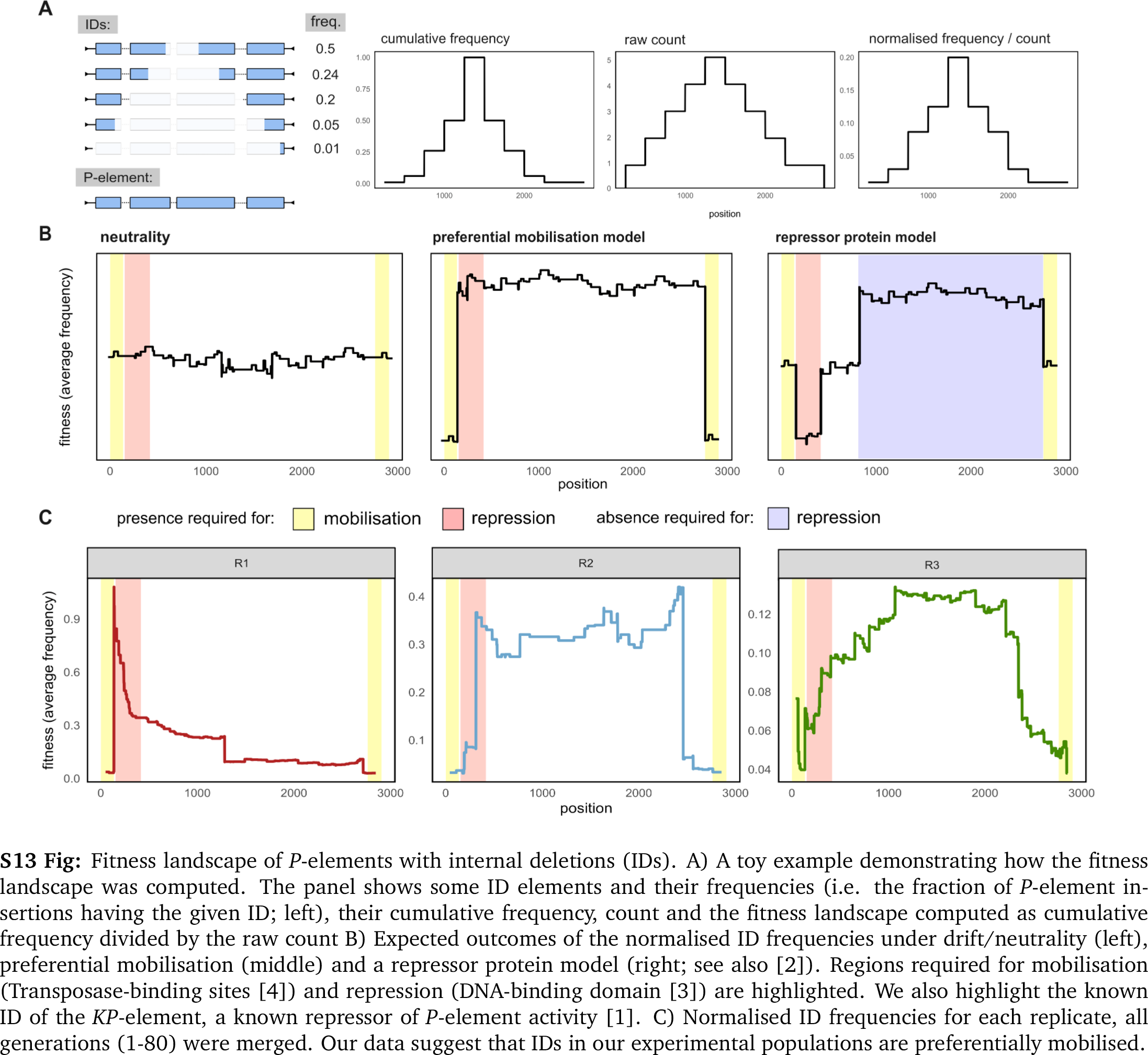

Supplement: S13 Fig — (TIF) [file pgen.1011649.s013.tif]

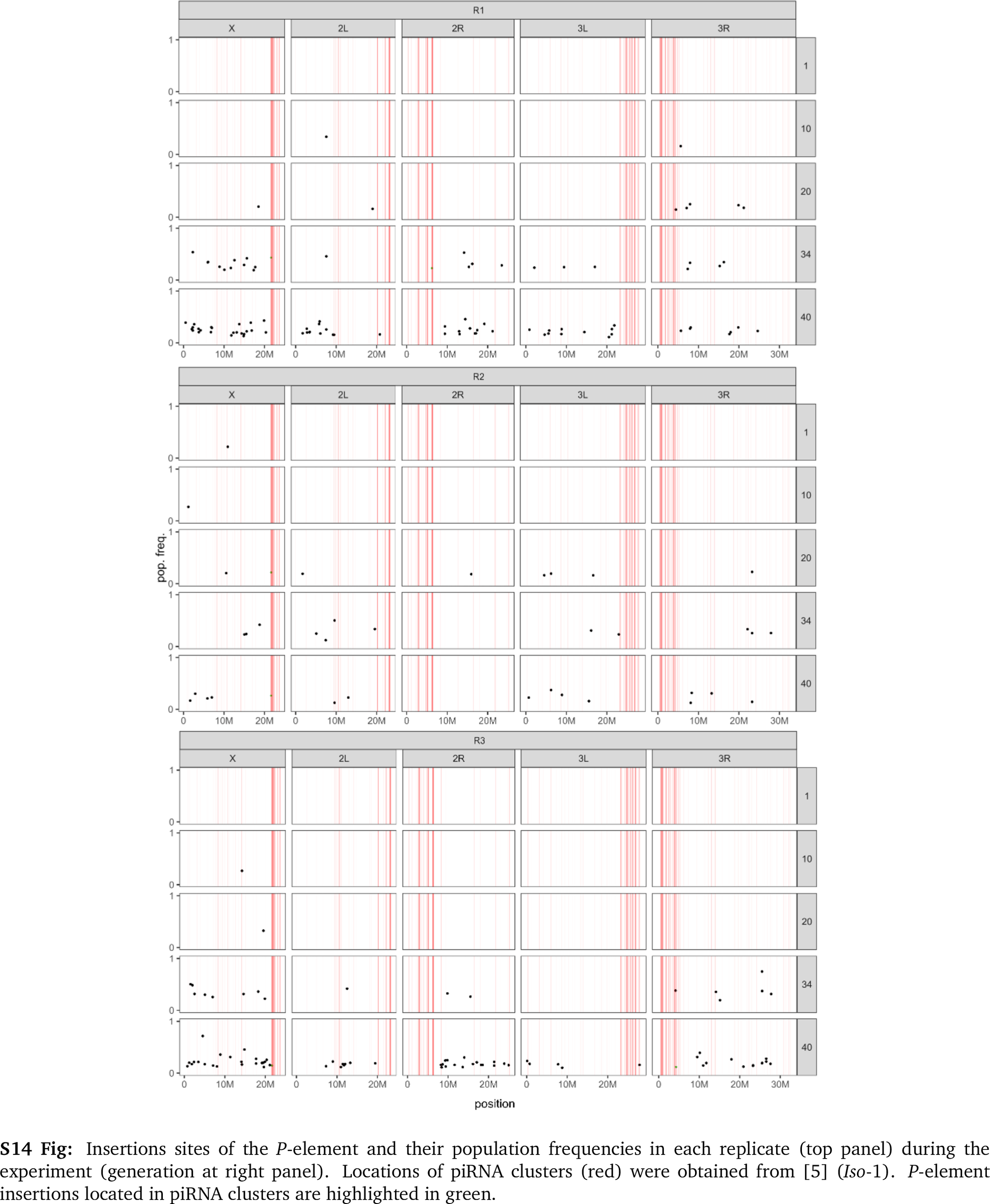

Supplement: S14 Fig — (TIF) [file pgen.1011649.s014.tif]
